# Co-occurring superior mesenteric artery syndrome and nutcracker syndrome requiring Roux-en-Y duodenojejunostomy and left renal vein transposition: a case report and review of the literature

**DOI:** 10.1186/s13256-018-1743-7

**Published:** 2018-08-06

**Authors:** Rebeca Heidbreder

**Affiliations:** PsychResearchCenter, LLC, 3669 Michaux Mill Drive, Powhatan, Virginia 23139 USA

**Keywords:** Superior mesenteric artery syndrome, Renal nutcracker syndrome, Left renal vein

## Abstract

**Background:**

The duodenum and the left renal vein occupy the vascular angle made by the superior mesenteric artery and the aorta. When the angle becomes too acute, compression of either structure can occur. Each type of compression is associated with specific clinical symptoms that constitute a rare disorder. If clinical symptoms are mild, conservative treatment is implemented. However, surgery is often the only solution that can improve quality of life and/or avoid life-threatening complications. This report describes a case of a patient with both types of aortomesenteric compression that required two separate surgeries to alleviate all symptoms.

**Case presentation:**

A 20-year-old white woman presented to the Emergency Room complaining of sudden onset severe left flank and lower left quadrant abdominal pain, nausea, and vomiting. A clinical work-up revealed elevated white blood cells and hematuria. She was discharged with a diagnosis of urinary tract infection. Symptoms continued to worsen over the subsequent 2 months. Repeated and extensive clinical work-ups failed to suggest evidence of serious pathology. Ultimately, an endoscopy revealed obstruction of her duodenum, and barium swallow identified compression by the superior mesenteric artery, leading to the diagnosis of superior mesenteric artery syndrome. She underwent a Roux-en-Y duodenojejunostomy. Six weeks later she continued to have severe left-sided pain and intermittent hematuria. Venography revealed compression of the left renal vein, extensive pelvic varices, and significant engorgement of her left ovarian vein. A diagnosis of nutcracker syndrome was made and a left renal vein transposition was performed. Significant improvement was seen after 8 weeks.

**Conclusions:**

The disorders associated with aortomesenteric compression can lead to serious symptoms and sometimes death. Diagnosis is challenging not only because of the lack of awareness of these rare disorders, but also because they are associated with symptoms that are similar to those seen in less serious diseases. Guidance for health care professionals with respect to relevant radiological and clinical markers needs to be reconsidered in order to clarify the etiology of the diseases and create better diagnostic protocols.

## Background

The superior mesenteric artery (SMA) arises from the anterior portion of the abdominal aorta behind the body of the pancreas and leaves the aorta usually at the level of the first lumbar vertebra approximately 1 cm below the origin of the coeliac trunk. The aortomesenteric angle created by the SMA and aorta is maintained by the left renal vein (LRV) crossing the vertebral column, the uncinate process of the pancreas, retroperitoneal lymphatic tissue, and a pad of mesenteric fat. The SMA also passes over the third part of the duodenum, which is suspended within the angle by the ligament of Treitz. The aortomesenteric distance is usually 10 to 28 mm and the downward angle of the SMA normally ranges from 38 to 65 degrees. This acute angulation is in contrast to the nearly complete right angle found in quadrupeds and is attributed to the erect posture of humans [1, 2].

Given that both the duodenum and the LRV lie within the aortomesenteric angle, two different types of compression syndrome can arise. The aortomesenteric compression of the duodenum is a rare radiological finding (< 1%) that is seen most commonly in females with an asthenic build and who are between the ages of 10 and 39 years [3–5]. In contrast, asymptomatic aortomesenteric compression of the LRV is a relatively common finding on computed tomography (CT; prevalence of between 51 and 72%) that is detected only slightly more frequently in females, and though the disorder is seen across a wide age range, it is most prevalent in young and middle-aged adults [6, 7].

The aortomesenteric compression syndrome of the duodenum was first described by Carl von Rokitansky in 1842 [8]. The first time the term “superior mesenteric artery syndrome” (SMAS) was used was in a publication by Kaiser *et al*. in 1960 [9]. To date, it remains the most often used term to describe the condition. In the more than 60 years since Goin and Wilk [5] stated that the condition was “somewhat more common than is generally appreciated …and [too often] it is so readily overlooked [that] patients suffering from it are often abandoned as neurotics, and what is actually a rare disorder becomes ...non-existent”, the diagnosis of SMAS still remains challenging and is not often considered part of the differential diagnosis in patients presenting with chronic or acute gastrointestinal (GI) symptoms [10–12].

SMAS consists chiefly of non-specific GI symptoms that include nausea, early satiety, abdominal pain, and vomiting, all of which are aggravated by eating [3, 11, 13, 14]. SMAS can be either intermittent/chronic [8, 15–19] or acute [8, 20–23], and physical and laboratory examinations often do not reveal any specific evidence that could aid in the diagnosis [21, 22, 24, 25]. In fact, approximately 40% of patients suffering from SMAS present with no obvious cause for having the disorder [1, 12, 26]. As a result, patients may either receive an incorrect diagnosis that delays their treatment, or correlations are made between SMAS and other disease processes that may not be valid [17, 27, 28].

Patients with a diagnosis of SMAS often have an asthenic build and are underweight [3, 10, 13, 20, 29]. Extreme weight loss is often suspected of causing the compression of the duodenum because it results in the loss of mesenteric fat which, in turn, decreases the aortomesenteric distance and leads to a more acute vascular angle [2, 3, 11, 13, 14, 30]. However, because SMAS is most often suspected once the patient has already lost weight, it is difficult to disambiguate if the weight loss causes the compression or if the compression causes the weight loss. If SMAS is caused by significant weight loss, one would assume that the frequency of SMAS would be higher in the population of individuals suffering from disorders that lead to significant weight loss such as anorexia nervosa; however, SMAS remains a rare complication of this disorder [31]. Furthermore, there exists a great variability in the percentage of patients diagnosed with SMAS who also experienced weight loss (25%–76%) [1, 12, 14], as well as evidence for no correlation between symptoms and body mass index (BMI) [3, 8, 12, 22, 23, 26, 32–37], and even cases of SMAS in obese patients [32, 38]. What is clear is that the anorexia and food aversion associated with SMAS lead to weight loss and a further aggravation of symptoms [13, 26, 30].

A recent report suggested that visceral fat volume does not correlate strongly with duodenal distance [39]. Normal patients with either low or high amounts of visceral fat can have either acute or wide SMA angles, and lack of fat does not always play a role in the initiation or resolution of SMAS [34, 40]. Furthermore, not only are the symptoms of SMAS intermittent, but radiographic findings associated with SMAS may also be intermittent and not related to weight loss [41]. Specifically, an occlusion will be most evident during an attack and could be moderately evident or even absent in the symptom-free period [24, 42, 43]. Peripheral parenteral nutrition to regain the weight loss with the aim of increasing mesenteric fat and lifting the SMA off the duodenum is very often unsuccessful, delays definitive treatment, and can lead to long-lasting serious complications [33, 38, 44]. In summary, although weight loss is very often seen in patients with SMAS, the role of weight loss in the initiation of the disease remains unclear.

SMAS has also been associated with “cast syndrome” because the diagnosis of SMAS is made following weight loss due to the immobilization of the patient by a body cast used to stabilize the spine after surgery to correct malformations of the spine [45]. It is now clear that not only a body cast, but any traction device that elongates the spine to correct a spinal deformity can produce a duodenal compression [46–48]. However, the correction of spinal deformity is neither a necessary nor a sufficient condition to provoke SMAS. Rather, the syndrome occurs with the same frequency (< 1%) in the population of patients who have corrective surgery of the spine as it does in other populations [4, 49]. Spinal corrective surgery can result in spinal lengthening which, in turn, can result in the cephalad displacement of the SMA [46, 50]. This directional change in position may be sufficient to create a more acute aortomesenteric angle and lead to a compression of the duodenum severe enough to produce clinical symptoms. Likewise, as an individual born with a congenital spinal or growth deformity continues to grow, he/she could end up with an acute aortomesenteric angle as the spine lengthens, resulting in a duodenal compression that will eventually precipitate the symptoms associated with SMAS [28, 50–54].

Surgeries that change the tension of the small bowel mesentery or the SMA itself may also change the aortomesenteric angle [55]. In addition, there is at least one report of resection of the uncinate process of the pancreas that resulted in SMAS ostensibly because of the role of the uncinate in maintaining the angle between the SMA and the aorta [56]. Finally, there are reports of SMAS following traumatic injury and brain injury [57, 58] as well as cases of individuals who suffered from a spinal injury and over time developed SMAS [51, 59, 60].

There may also be a congenital explanation for SMAS that is supported by reports of SMAS in neonates [61, 62], identical twins [26], and within families [35, 63]. It may be the case that a higher positioned duodenum within a rather more acute aortomesenteric angle may leave enough room for the duodenum during childhood, but as the duodenum grows with age the available space within the angle becomes insufficient [1]. The duodenum fits so exactly into the vascular angle that normal variations of the aortomesenteric angle, the level at which the duodenum crosses the vertebral column, the length and attachment of the ligament of Treitz, or the degree of lumbar spinal curvature may all predispose to duodenal compression. Furthermore, any increase in lordosis with age could also increase the possibility of compression [1, 5]. Thus, it is clear that any factor, not only weight loss, that decreases the space around the duodenum or in some way alters its relationship to the surrounding anatomical structures increases the probability of external compression or occlusion of the duodenum [3, 10, 13].

Belief that weight loss causes or at the very least worsens the symptoms of SMAS, leads to a tendency to opt for conservative treatment. This often means hyperalimentation either through small meals, enteral, or even parenteral nutrition. Although there is evidence for a successful outcome using these methods [11, 13, 19, 27, 29, 36, 64], there are very little follow-up data reporting the duration of the efficacy of the treatment. The fact that a patient has minimal weight gain (2–3 kg) with coincident improvement in symptoms does not guarantee a non-recurrence of symptoms, particularly since SMAS is well known to be intermittent in many patients [8, 10, 15–19]. A “wait and see” approach with aided nutrition may actually provoke more serious symptoms in the long run, including an excessively enlarged stomach and gastroparesis [65–67]. A delay in diagnosis and/or appropriate treatment increases the probability of a less favorable prognosis and in some cases can be life-threatening [12, 25, 28, 38, 42, 44, 68, 69].

One study that followed 80 patients reported a greater success rate in patients treated surgically than in patients treated medically both in terms of outcome and recurrence [12]. The most common surgery used to treat SMAS since at least 1912 is the duodenojejunostomy via laparotomy [37, 44, 69, 70]. More recently, the duodenojejunostomy has been performed laparoscopically with mixed results [12, 23, 30]. Gastrojejunostomy [71], duodenal derotation [72], and duodenal circular drainage [73] as well as the division of the ligament of Treitz [74] have also been tried with limited success. Most recently, transposition of the SMA itself has had positive results [75–77], although the inherent risks with the procedure make it impractical in all but the most expert hands. Irrespective of the surgical procedure, and as with conservative therapy, what is still lacking is appropriate patient follow-up in order to understand the actual long-term success rate of any procedure.

Nearly one century after the discovery that the duodenum could be compressed between the SMA and the aorta, the anatomist Grant made the first mention that the LRV could also be trapped beneath the SMA [78]. The first clinical report of the condition was made by El-Sadr and Mina in 1950 [79]. In 1972 the Belgian physician, de Schepper, named the condition nutcracker syndrome (NCS) [80]. Sometimes NCS and another term nutcracker phenomenon (NCP) are used interchangeably. However, the clinical diagnosis of NCS is usually reserved for those patients who have evidence not only of LRV stenosis, but also exhibit specific clinical symptoms. In contrast, NCP is used to refer to the asymptomatic morphologic compression of the LRV between the SMA and aorta [81–83] and is a fairly common incidental finding on abdominal CT [84].

As is the case for SMAS, decreased aorta–SMA distance leads to LRV entrapment. However, many individuals with abnormally acute aortomesenteric angles have no signs of the disorder [83, 85, 86]. Although patients with NCS can present with weight loss and lower BMI [81, 86], many more have a normal BMI [87, 88]. The main clinical manifestations associated with NCS are hematuria (78.5%) and pelvic or flank pain (36%). The hematuria is believed to be the result of an increase in pressure within the LRV that weakens its walls, leading to the predisposition for rupture of the septum between the small veins and the collecting system [6, 7, 81, 87, 89]. Hematuria is most often microscopic, and when macroscopic (< 20% of patients) is also often intermittent [81, 90]. The flank pain has been attributed to inflammatory processes secondary to the LRV constriction or urethral colic with the passage of blood clots [6, 82, 87]. It may also be related to the engorgement of the left gonadal vein due to the constricted blood flow between the gonadal vein and the LRV [83, 87, 91–93]. Approximately 30% of patients also have proteinuria, which increases the probability of reaching the diagnosis of NCS. The proteinuria can reach very high levels (> 400 mg/dl), particularly when the patient has been standing for more than 15 minutes [7, 90]. Pelvic congestion is seen in up to 83% of female patients with LRV compression [83, 91–94], but can occur as a result of many other factors and even asymptomatic LRV stenosis [83, 89, 94]. As with SMAS, the diagnosis of NCS is difficult because of the similarity of symptoms with other diseases, especially nephrolithiasis, urinary tract infection (UTI), or severe menstrual cramps. As such, patients most often must undergo numerous investigations, which can range from months to years, before a diagnosis is made [93].

Although LRV compression as well as gonadal vein engorgement can be readily identified on CT scan, confirmation of an NCS diagnosis can only be made with renal angiography or LRV venography such that not only the compression, but also the increased pressure gradient of greater than 3 mm Hg between LRV and inferior vena cava (IVC) can be established [82, 95]. Marked clinical symptoms arise only when the LRV stenosis becomes hemodynamically significant and leads to venous hypertension [89]. It bears noting, however, that there is no clear consensus for a definitive cut-off value for NCS diagnosis given that the renocaval pressure gradient in normal patients can vary between 4 and 7 mm Hg, and those in patients with NCS can range between 3.6 and 10.5 mm Hg. In addition, in patients with chronic LRV compression, the associated gonadal vein reflux and formation of pelvic varices may result in a lower pressure gradient, but an increased renal venous baseline [96]. In sum, unlike SMAS, NCS is not primarily a radiologic diagnosis, but rather one that must also tie in clinical and laboratory data.

Several surgical procedures have been tried in patients with a confirmed diagnosis of NCS and whose symptoms are severe enough to warrant a surgical solution. LRV transposition, involving transection of the LRV and moving it distally on the IVC where it is re-anastomosed, is the most frequently performed surgery to treat the condition [85, 87, 97–100]. Although decrease of pelvic congestion and/or gonadal vein engorgement can be obtained by left gonadal vein transposition or ligation, this procedure is not recommended without the appropriate resolution of LRV compression because of the possible increase in the renocaval pressure gradient [96, 101, 102]. Other surgical procedures for the treatment of NCS include endovascular stenting [92, 103, 104], gonadocaval bypass [95], gonadal vein transposition [101, 105], saphenous vein bypass [106], and renal autotransplantation and nephrectomy [85, 97, 107].

There are only a few reported cases in the literature that describe patients with radiological evidence of the compression of both the duodenum and the LRV [2, 108–113], and there is only one report of a patient who had both the radiological and clinical evidence for both disorders [112]. The case presented in this report is unique in that it is the first in the literature to describe a patient who was diagnosed as having both SMAS and NCS, and who required two separate surgeries to resolve all clinical symptoms.

## Case presentation

A 20-year-old white woman arrived at the Emergency Room (ER) complaining of sudden onset severe left flank and lower left quadrant (LLQ) abdominal pain, nausea, and vomiting. Her height and body weight were 180 cm and 63.5 kg (BMI of 19.5). Her history revealed that at the onset of pain, she believed she was suffering from severe menstrual cramps. The pain was not relieved by non-steroidal anti-inflammatory drugs (NSAIDs) even at higher doses. Eventually she became nauseated and started vomiting. She admitted to having felt increasingly more nauseated for several months prior, but had not vomited until the day she arrived at the ER. Her past clinical history included type IV (Graf classification) congenital bilateral developmental dysplasia of the hip diagnosed at birth (now resolved), adenoidectomy (3 years of age), and severe menstrual pain starting at 15 years of age, which had increased in severity over the course of the subsequent 4 years. There was no other remarkable clinical history, injury, or accident.

She was afebrile, and laboratory results were unremarkable with the exception of a white blood cell (WBC) count of 13 and gross hematuria with significant WBC in her urine. On physical examination, her abdomen was very tender in her left flank, LLQ, and pelvic area. She denied burning during urination and frequency. A pelvic ultrasound (US) was read as unremarkable. No other tests were ordered. She was released with the diagnosis of cystitis/UTI and prescribed ciprofloxacin, ibuprofen, oxycodone, and ondansetron.

Four days later she returned to the ER complaining once again of severe abdominal pain, but now also vomiting violently. The pain was no longer localized to just her left flank and LLQ, but had generalized to her right upper quadrant (RUQ) and periumbilical region. Repeat bloodwork revealed that WBC was now 10, but serum amylase was 220 and lipase was 120. Urine analysis still showed some red blood cells (RBC) and WBC. A CT with intravenously administered contrast was ordered and was read as unremarkable. An abdominal US showed a left-sided kidney stone. A pelvic US showed a right-sided ovarian cyst. A US of her gall bladder revealed no calculi or pathology.

Three days after passing the kidney stone, she had an increased feeling of swelling, pressure, and pain in her left flank. A ureteroscopy was performed to confirm the absence of any pathology. Her left ureter had minimal reflux, but otherwise no abnormalities were noted. A second CT scan was performed, but again no obvious pathology was noted.

With no improvement 2 weeks later, the decision was made to perform an exploratory laparoscopy, which revealed severe pelvic congestion as well as small amounts of endometriosis. Her appendix was thickened and had an appendicolith and was removed. Repeat bloodwork showed that her amylase and lipase levels had returned to normal. She was discharged the same day.

Two weeks after the laparoscopy when there was still no improvement in the pain, nausea, and anorexia and because she started experiencing post-prandial satiety, an endoscopy was performed. It revealed a moderate dilation of her duodenum as well as fluid in her stomach despite the fact that she had not eaten or drunk for more than 24 hours. The findings from the endoscopy prompted a review of the images from the initial CT, and this time it was determined that her duodenum was being compressed by an external structure. An enterography and a barium swallow confirmed the constriction of the duodenum by the SMA, and she was finally given the diagnosis of SMAS. Despite the fact that she had already suffered a net weight loss of 8 kg, the option to treat the disorder conservatively by enteral or parenteral feeding was not considered since she was of normal weight when the symptoms started and there was little expectation that weight gain would resolve the issue. She underwent a Roux-en-Y duodenojejunostomy. An iodine swallow 3 days post-surgery showed that fluid moved freely through the anastomosed areas. She was discharged 4 days later, able to tolerate soft foods.

Four weeks after the duodenojejunostomy, the pain in her left flank became even more severe, and she also started experiencing urinary hesitancy as well as pain in both flanks and her pelvic region during urination and bowel movements. The severity of the pain in her left flank increased even more during the week of her menstrual cycle. Another careful review of the very first CT scan performed at the beginning of the onset of symptoms led to the conclusion that her SMA was also compressing her LRV, her left ovarian vein was engorged, and the severe pelvic congestion, initially discovered during the laparoscopy, was also visible. A selective venography of her LRV showed significant stenosis of the LRV, a markedly enlarged ovarian vein with retrograde flow into her pelvis, and multiple enlarged pelvic veins. The pressure gradient between the renal side of the vein and the IVC side of the vein was 5 mm Hg. These findings established the diagnosis of NCS.

Eleven weeks after the duodenojejunostomy, she underwent an LRV transposition in which her adrenal vein and the ovarian vein were also tied off. Following this second surgery she began to improve considerably. One month after surgery her left flank and RUQ pain were significantly decreased, and though the nausea persisted, she was able to eat without vomiting and started to regain the weight she lost.

Eight months after the Roux-en-Y duodenojejunostomy and 4 months after the LRV transposition, a CT showed patent anastomoses, reduction in the number of pelvic varices, and reduced diameter of her ovarian vein. She was able to eat with minimal GI disturbance and had regained 5 kg.

## Discussion

The idea that the SMA could be the culprit behind a compression syndrome involving the duodenum was first suggested in 1842 [8]. Since then the literature for the SMA compression of the duodenum has waxed and waned for more than a century and a half due in part to the fact that the disorder seems to become endemic only in areas where radiologists are aware of and trained in its diagnosis. However, what has come to be accepted is that SMA compression of the duodenum is a rare disorder [4, 5] that affects females more than males, can be evident at any age, but is seen most frequently in patients between the ages of 10 and 39 [12], and is often associated with individuals who have lean or underweight body habitus [3, 5].

Grant [78] established that the SMA can also compress the LRV. This compression is only slightly more biased toward females, has no real age band, and there is no consistent association with the weight of the patient compared with patients in whom the compression involves the duodenum. Furthermore, although compression of the LRV is a common radiological finding, the frequency with which the compression actually causes pathological symptoms remains unclear. It is believed, however, that the manifestation of pathological symptoms constitutes a rare disorder [81, 84, 98].

Given that both the duodenum and the LRV occupy a place within the same vascular angle, and that the compression of either structure is contingent upon a vascular angle that is significantly below the normal limit (< 38 degrees), it is curious that a double compression has been reported only a few times in the literature [2, 108–113]. Although the aortomesenteric compression of the duodenum has often been widely attributed to excessive weight loss, the same argument has not been consistently made for the aortomesenteric compression of the LRV. This may be due to the difference in the expectation of symptoms, which in the case of SMAS include anorexia and vomiting, and weight loss, but NCS produces urinary and/or gynecological symptoms but generally no weight loss. NCS and SMAS also differ greatly in terms of the frequency of incidental findings of a compression. The significantly greater number of LRV compressions indicate that an aortomesenteric angle outside the normal range can be a preexisting condition even before producing clinical symptoms. The absence of clinical symptoms in the presence of radiological evidence of LRV compression can be explained in part by the vascular system’s ability to create compensatory strategies in the form of collaterals to overcome the occlusion. As a result, true clinical symptoms may not be noted until the circulatory system reaches a state of excessive hydrostatic pressure. However, the same is not true for the GI system. When there is an intestinal occlusion, clinical symptoms become evident almost immediately because the only compensatory mechanisms involve the rejection of ingested material in ways that are externally obvious. As such, the weight loss noted in many patients may be the end result of the occlusion and not the precipitating factor. The weight loss may simply provoke a further narrowing of a preexisting abnormally acute aortomesenteric angle, and, as a result, the vomiting and compression become self-perpetuating regardless of the initiating factors [40], particularly since we know that the compression can progress from irrelevant to complete obstruction [3, 72]. Evidence of SMAS in individuals with certain skeletal deformities or who have undergone a surgical intervention to correct a skeletal problem suggests that rather than weight loss, certain congenital anatomic factors or post-surgical changes in skeletal conformation predispose an individual to this disorder [3, 5, 26, 28, 50–54, 61–63].

NCS is a historically more recent clinical phenomenon than SMAS. This may be due in part to the less salient symptoms associated with NCS compared to SMAS as well as the higher frequency of asymptomatic LRV compression both of which give the disorder less clinical notoriety. Whereas the precipitating factor or factors that may lead to clinical symptoms in SMAS remain debatable, cases of LRV compression that become clinically significant are most likely due to significant changes in the hydrostatic pressure in the LRV and the compensatory collaterals. In other words, whereas the pressure increase produced by the compression may initially be relieved by the formation of collaterals and the retrograde flow into the gonadal vein and ectasia of pelvic vessels, it is only when the compensatory mechanisms fail or become overloaded that the patient begins to exhibit symptoms. For this reason, LRV compression may be a smoldering undetected issue for years, and, as is often the case in SMAS, once the clinical symptoms become severe enough to warrant medical intervention, patients may still be misdiagnosed or treated simply for pain due to the lack of awareness about the disease space.

A first step toward a greater understanding of either type of compression and/or their interrelation is to intentionally include these disorders as part of the differential diagnosis when reading radiographic images for those individuals presenting with the clinical symptoms associated with either of these disorders. Equally important is the interdisciplinary collaboration between the areas of medicine that will be the first identifiers of the disorders, namely ER, radiology, GI, urology, and gynecology and perhaps even orthopedics.

In addition, the systematic documentation, either as a retrospective review of abdominal CTs or an intentional cataloging of CTs going forward, of the number of individuals who have one or both compressions even in the absence of clinical symptoms is necessary and warranted. This kind of documentation is important for several reasons. First, since the identification of a compression will become part of a patient’s medical history especially in the absence of current clinical symptoms, it would help to answer the question of what additional factor or factors have to be present for one or both of the compressions to precipitate clinical symptoms. Second, it would clarify the role of the LRV in maintaining the aortomesenteric angle. Third, it would provide information regarding the importance of the position of the duodenum within the aortomesenteric angle, particularly given the fact that exponentially more patients have LRV than duodenal compression despite the fact that the LRV plays a role in maintaining the aortomesenteric angle [3].

## Conclusions

Currently, the diagnosis of either type of aortomesenteric compression disorder remains complicated despite the fact that they are easily detected on CT scan with intravenously administered contrast. Although a positive finding on CT does not provide the absolute diagnosis, evidence of a compression does provide the physician with sufficient information to warrant further testing. However, despite the information provided by CT, the diagnosis of either compression disorder often escapes the diagnostic focus of the physician no doubt due in part to the lack of notoriety of the disorders, but also in part due to the reality that medical professionals need to be parsimonious and efficient and, as a result, valuable information present in imaging studies is often glossed over. In the end, failure to diagnose or a misdiagnosis can end up being more expensive in terms of financial burden to an individual or society, as well as a psychological burden to the patient who does not obtain the relief that is commensurate with his or her condition.

GI symptoms are not common in cases of LRV compression; therefore, the presence of such symptoms in patients diagnosed with NCS should alert the physician to the possibility of a double compression, prompting the need for further investigation. The reverse necessarily must also be true. A patient, who first presents with radiographic and clinical evidence of SMAS, could also simultaneously or sometime thereafter present with clinical symptoms that point to a urologic or gynecologic dysfunction. In this case, the possibility of a compression of the LRV should be ruled out. The diagnosis should be further suspected if there is evidence of pelvic congestion, varicocele and/or engorgement of the gonadal vein, all of which are particularly remarkable findings in younger and nulliparous patients.

Abdominal pain, particularly in young women, is one of the most common complaints seen in the ER, and all too often patients who do not exhibit a constellation of symptoms that are pathogenetic, ultimately end up with an idiopathic diagnosis. Equally often, the ailing patient is relegated to the category of “psychosomatic” and dismissed and left to suffer or prescribed narcotics that could lead to devastating consequences with prolonged use. With the advent of and widespread use of social media, it is easy to find evidence of both of these diseases on platforms such as Facebook and Instagram that describe the long and painful histories of individuals who were undiagnosed, misdiagnosed, or treated inappropriately.

In sum, a more complete understanding of these disease spaces will require further research. To start, future meta-analyses of abdominal CT scans need to be conducted in order to determine the true frequency of the compression with and without symptoms. In addition, protocols should be put in place to include both types of compressions in the standard differential diagnosis when abdominal CT scans are performed. Not to do so is simply a waste of valuable information. Even if the patient is not exhibiting clinical symptoms at the moment of the CT scan, the compression(s) should be noted as part of the patient’s medical history, particularly given the intermittent character of both disorders, the possibility of progression of the compression, and the lack of understanding related to what factors may precipitate acute symptoms.

## Abbreviations

BMI: Body mass index
CT: Computed tomography
ER: Emergency Room
GI: Gastrointestinal
IVC: Inferior vena cava
LLQ: Lower left quadrant
LRV: Left renal vein
NCP: Nutcracker phenomenon
NCS: Nutcracker syndrome
NSAID: Non-steroidal anti-inflammatory drug
RBC: Red blood cell
RUQ: Right upper quadrant
SMA: Superior mesenteric artery
SMAS: Superior mesenteric artery syndrome
US: Ultrasound
UTI: Urinary tract infection
WBC: White blood cell
